# SEROTONIN SIGNALING IN HOST-PATHOGEN RESPONSES

**DOI:** 10.1093/geroni/igaf122.1797

**Published:** 2025-12-31

**Authors:** Sean Curran, Tripti Nair

## Abstract

When an organism encounters a pathogen, the host innate immune system activates to defend against pathogen colonization and toxic xenobiotics produced. C. elegans employ multiple defense systems to ensure survival when exposed to Pseudomonas aeruginosa including activation of the cytoprotective transcription factor SKN-1/NRF2. Although wildtype C. elegans quickly learn to avoid pathogens, here we describe a peculiar behavior in animals with constitutive activation of SKN-1 whereby they choose not to leave the pathogenic environment even when a non-pathogenic and healthspan-promoting food options are available. SKN-1 activation, specifically in neurons and intestinal tissues, orchestrates a unique transcriptional program which leads to defects in serotonin signaling that is required from both neurons and non-neuronal tissues. Serotonin depletion from SKN-1 activation limits pathogen defenses capacity, drives the pathogen-associated apathy behaviors and induces a synthetic sensitivity to selective serotonin reuptake inhibitors. We further define the serotonin receptors required for this behavioral response to Pseudomonas. Taken together, our work reveals interesting insights into how animals perceive environmental pathogens and subsequently alter behavior and cellular programs to promote survival.

